# An Improved Interacting Multiple Model Filtering Algorithm Based on the Cubature Kalman Filter for Maneuvering Target Tracking

**DOI:** 10.3390/s16060805

**Published:** 2016-06-01

**Authors:** Wei Zhu, Wei Wang, Gannan Yuan

**Affiliations:** College of Automation, Harbin Engineering University, No. 145 Nantong Street, Harbin 150001, China; zhuwei_heu@163.com (W.Z.); yuangannan@163.com (G.Y.)

**Keywords:** target tracking, cubature Kalman filter, unscented Kalman filter, interacting multiple models

## Abstract

In order to improve the tracking accuracy, model estimation accuracy and quick response of multiple model maneuvering target tracking, the interacting multiple models five degree cubature Kalman filter (IMM5CKF) is proposed in this paper. In the proposed algorithm, the interacting multiple models (IMM) algorithm processes all the models through a Markov Chain to simultaneously enhance the model tracking accuracy of target tracking. Then a five degree cubature Kalman filter (5CKF) evaluates the surface integral by a higher but deterministic odd ordered spherical cubature rule to improve the tracking accuracy and the model switch sensitivity of the IMM algorithm. Finally, the simulation results demonstrate that the proposed algorithm exhibits quick and smooth switching when disposing different maneuver models, and it also performs better than the interacting multiple models cubature Kalman filter (IMMCKF), interacting multiple models unscented Kalman filter (IMMUKF), 5CKF and the optimal mode transition matrix IMM (OMTM-IMM).

## 1. Introduction

Bayes filtering algorithms have been broadly used in target tracking systems [1,2,3,4], while a large number of Gaussian approximation filters and Monte Carlo filters have been introduced to solve target tracking problems [5]. Although the particle filter (PF) can deal with non-linear and non-Gaussian systems, the computational complexity always makes its use prohibitive [6]. Gaussian approximation filtering algorithms are more efficient. Among the Gaussian approximation filters, the extended Kalman filter (EKF) has been widely used in nonlinear systems [7,8]. It uses first order Taylor series expansion, which can induce deviations when the systems have higher order and complex non-linear character. In order to reduce the system linearization errors, the unscented Kalman filter (UKF) was introduced to deal with nonlinear systems and it outperforms EKF [9]. Recently, Arasaratnam and Haykin presented the cubature Kalman filter (CKF) based on the spherical-radial cubature rule [10,11]. The CKF has a rigid mathematical proof that is different from the UKF, and both UKF and CKF can approximate the model of the system using specially chosen points. It has been proved that when the dimension of the system is three, the CKF has the same performance as the UKF [12].

Blom and Shalom have proposed the interacting multiple model (IMM) algorithm based on a generalized pseudo-random algorithm to decrease the error of single model algorithm, which will improve the quick response and accuracy of target tracking [13]. The IMM algorithm processes all the models simultaneously and changes different models by checking their weights. It has been proved that the IMM algorithm performs better than any single model algorithm in complex tracking problems [14]. Many filters have been integrated with the IMM algorithm to enhance the accuracy and quick response of nonlinear target tracking [14,15,16]. The performance of interacting multiple models unscented Kalman filter (IMMUKF) is compared with the interacting multiple models extended Kalman filter (IMMEKF), and the results show that IMMUKF performs better than IMMEKF in bearings-only maneuvering tracking problems [15]. However, when the dimension of the system is more than three, the weights of UKF are negative which will cause the divergence of the filter [12,17]. Then the CKF is introduced in IMM to overcome the issue, and the new algorithm can reduce the computational complexity and improve the accuracy of the filter [17,18]. Lee, Motai and Choi have proposed the multichannel interacting multiple model estimator (MC-IMME) to improve the overall performance of the traditional particle filter, ensemble KF and IMME [19]. The multiple delta quaternion extended Kalman filter is proposed in [20] for head orientation prediction. The proposed multiple model delta quaternion (DQ) (MMDQ) filters integrate constant velocity (CV) and constant acceleration (CA) DQ filters in an IMME framework, and the experimental results show that the new filter performs better than DQ-EKF albeit with increased computation. In [21], the authors proposed a sensor fusion algorithm which introduces dynamic noise covariance matrix into interacting multiple models. The proposed filter is more accurate than the Kalman filter when there are abrupt changes in the path of the vehicle. In order to improve the accuracy of the traditional IMM algorithm, the optimal mode transition matrix IMM (OMTM-IMM) algorithm was proposed in [22]. The OMTM-IMM utilizes the linear minimum variance theory to minimize the error of the initial state and the simulation results show that it outperforms the traditional IMM when the sojourn times of the system are not known.

In this paper, the interacting multiple models five degree cubature Kalman filter (IMM5CKF) based on a five degree cubature Kalman filter and IMM algorithm is proposed to improve the tracking accuracy, model estimation accuracy and quick response of target tracking algorithms. The negative weights of 5CKF go to 0 when the system dimensions go to ∞, so 5CKF is more stable than UKF [23]. The simulation results show that the IMM5CKF exhibits better accuracy and switching sensitivity performance than IMMCKF, IMMUKF, 5CKF and OMTM-IMM. The remainder of the paper is organized as follows: the high degree of cubature Kalman filter is analyzed in Section 2. In Section 3, IMM5CKF is derived. The performance of the target tracking algorithms are compared in a benchmarked target tracking problem in Section 4. Conclusions are given in Section 5.

## 2. Five Degree Cubature Kalman Filter

The five degree cubature Kalman filter is proposed to improve the accuracy of the traditional Cubature Kalman Filter [23]. It chooses deterministic odd points to transfer the nonlinear functions to calculate the posterior mean and covariance of the system.

Supposing state variables $x=N\left(\bar{x},P\right)$, where $\bar{x}$ is mathematical expectation of *x*, *P* is the covariance of *x*. The five degree Cubature Kalman Filter includes two steps, time update and measurement update.

### 2.1. Time Update

(1) Factorize

The Cholesky decomposition of $P_{k-1|k-1}$ is calculated as:

(1) $$P_{k-1|k-1}=S_{k-1|k-1}S_{k-1|k-1}^{T}$$

(2) Evaluate the cubature points:

(2) $$X_{0i,k-1|k-1}={\hat{x}}_{k-1|k-1}$$

(3) $$X_{1i,k-1|k-1}=\sqrt{\left(n+2\right)}S_{k-1|k-1}e_{i}+{\hat{x}}_{k-1|k-1}$$

(4) $$X_{2i,k-1|k-1}=-\sqrt{\left(n+2\right)}S_{k-1|k-1}e_{i}+{\hat{x}}_{k-1|k-1}$$

(5) $$X_{3i,k-1|k-1}=\sqrt{\left(n+2\right)}S_{k-1|k-1}s_{i}^{+}+{\hat{x}}_{k-1|k-1}$$

(6) $$X_{4i,k-1|k-1}=-\sqrt{\left(n+2\right)}S_{k-1|k-1}s_{i}^{+}+{\hat{x}}_{k-1|k-1}$$

(7) $$X_{5i,k-1|k-1}=\sqrt{\left(n+2\right)}S_{k-1|k-1}s_{i}^{-}+{\hat{x}}_{k-1|k-1}$$

(8) $$X_{6i,k-1|k-1}=-\sqrt{\left(n+2\right)}S_{k-1|k-1}s_{i}^{-}+{\hat{x}}_{k-1|k-1}$$

(9) $$s_{i}^{+}=\left\{\sqrt{\frac{1}{2}}\left(e_{j}+e_{l}\right)\right\}:j<l,j,l=1,2,...,n$$

(10) $$s_{i}^{-}=\left\{\sqrt{\frac{1}{2}}\left(e_{j}-e_{l}\right)\right\}:j<l,j,l=1,2,...,n$$

(11) $$w_{0}=\frac{2}{n+2}$$

(12) $$w_{1}=\frac{4-n}{2{\left(n+2\right)}^{2}},i=1,2,\cdots n$$

(13) $$w_{2}=\frac{2}{{\left(n+2\right)}^{2}},i=1,2,...,n(n-1)/2$$

where $w_{0}$, $w_{1}$ and $w_{2}$ is the weights of the cubature points, $e_{i}$ is the unit vector.

(3) Evaluate the propagated cubature points

The sample points are obtained by propagating the cubature points through the state equation as:

(14) $$X_{0i,k|k-1}^{*}=f\left(X_{0i,k-1|k-1}\right)$$

(15) $$X_{1i,k|k-1}^{*}=f\left(X_{1i,k-1|k-1}\right)$$

(16) $$X_{2i,k|k-1}^{*}=f\left(X_{2i,k-1|k-1}\right)$$

(17) $$X_{3i,k|k-1}^{*}=f\left(X_{3i,k-1|k-1}\right)$$

(18) $$X_{4i,k|k-1}^{*}=f\left(X_{4i,k-1|k-1}\right)$$

(19) $$X_{5i,k|k-1}^{*}=f\left(X_{5i,k-1|k-1}\right)$$

(20) $$X_{6i,k|k-1}^{*}=f\left(X_{6i,k-1|k-1}\right)$$

(4) Estimate the predicted points

State prediction ${\hat{x}}_{k|k-1}$ is then calculated by the weighted combination of sample points as:

(21) $${\hat{x}}_{k|k-1}=w_{0}X_{0i,k|k-1}^{*}+w_{1}X_{1}^{*}+w_{2}X_{2}^{*}$$

(22) $$X_{1}^{*}=\sum_{i=1}^{n}(X_{1i,k|k-1}^{*}+X_{2i,k|k-1}^{*})$$

(23) $$X_{2}^{*}=\sum_{i=1}^{n(n-1)/2}(X_{3i,k|k-1}^{*}+X_{4i,k|k-1}^{*}+X_{5i,k|k-1}^{*}+X_{6i,k|k-1}^{*})$$

(5) Estimate the predicted error covariance:

(24) $$P_{k|k-1}=w_{0}X_{oi,k|k-1}^{*}X_{oi,k|k-1}^{*T}+P_{1}+P_{2}$$

(25) $$P_{1}=w_{1}\sum_{i=1}^{n}\left(X_{1i,k|k-1}^{*}X_{1i,k|k-1}^{*T}+X_{2i,k|k-1}^{*}X_{2i,k|k-1}^{*T}\right)$$

(26) $$P_{2}=w_{2}\sum_{i=1}^{n(n-1)/2}\left(X_{3i,k|k-1}^{*}X_{3i,k|k-1}^{*T}+X_{4i,k|k-1}^{*}X_{4i,k|k-1}^{*T}+X_{5i,k|k-1}^{*}X_{5i,k|k-1}^{*T}+X_{6i,k|k-1}^{*}X_{6i,k|k-1}^{*T}\right)$$

### 2.2. Measurement Update

(1) Factorize:

(27) $$P_{k|k-1}=S_{k|k-1}S_{k|k-1}^{T}$$

(2) Evaluate the cubature points:

(28) $$X_{0i,k|k-1}={\hat{x}}_{k|k-1}$$

(29) $$X_{1i,k|k-1}=\sqrt{\left(n+2\right)}S_{k|k-1}e_{i}+{\hat{x}}_{k|k-1}$$

(30) $$X_{2i,k|k-1}=-\sqrt{\left(n+2\right)}S_{k|k-1}e_{i}+{\hat{x}}_{k|k-1}$$

(31) $$X_{3i,k|k-1}=\sqrt{\left(n+2\right)}S_{k|k-1}s_{i}^{+}+{\hat{x}}_{k|k-1}$$

(32) $$X_{4i,k|k-1}=-\sqrt{\left(n+2\right)}S_{k|k-1}s_{i}^{+}+{\hat{x}}_{k|k-1}$$

(33) $$X_{5i,k|k-1}=\sqrt{\left(n+2\right)}S_{k|k-1}s_{i}^{-}+{\hat{x}}_{k|k-1}$$

(34) $$X_{6i,k|k-1}=-\sqrt{\left(n+2\right)}S_{k|k-1}s_{i}^{-}+{\hat{x}}_{k|k-1}$$

(3) Evaluate the propagated cubature points

The sample points are obtained by propagating the cubature points through the observation equation:

(35) $$Z_{0i,k|k-1}=h\left(X_{0i,k|k-1}\right)$$

(36) $$Z_{1i,k|k-1}=h\left(X_{1i,k|k-1}\right)$$

(37) $$Z_{2i,k|k-1}=h\left(X_{2i,k|k-1}\right)$$

(38) $$Z_{3i,k|k-1}=h\left(X_{3i,k|k-1}\right)$$

(39) $$Z_{4i,k|k-1}=h\left(X_{4i,k|k-1}\right)$$

(40) $$Z_{5i,k|k-1}=h\left(X_{5i,k|k-1}\right)$$

(41) $$Z_{6i,k|k-1}=h\left(X_{6i,k|k-1}\right)$$

(4) Estimate the predicted measurement:

(42) $${\hat{z}}_{k|k-1}=w_{.0}Z_{0i,k|k-1}+w_{1}Z_{1}+w_{2}Z_{2}$$

(43) $$Z_{1}=\sum_{i=1}^{n}\left(Z_{1i,k|k-1}+Z_{2i,k|k-1}\right)$$

(44) $$Z_{2}=\sum_{i=1}^{n(n-1)/2}\left(Z_{3i,k|k-1}+Z_{4i,k|k-1}+Z_{5i,k|k-1}+Z_{6i,k|k-1}\right)$$

(5) Estimate the innovation covariance matrix:

(45) $$P_{zz,k|k-1}=w_{0}Z_{0i,k|k-1}Z_{0i,k|k-1}^{T}+P_{zz1}+P_{zz2}-{\hat{z}}_{k|k-1}{\hat{z}}_{k|k-1}^{T}+R_{k}$$

(46) $$P_{zz1}=w_{1}\sum_{i=1}^{n}\left(Z_{1i,k|k-1}Z_{1i,k|k-1}^{T}+Z_{2i,k|k-1}Z_{2i,k|k-1}^{T}\right)$$

(47) $$P_{zz2}=w_{2}\sum_{i=1}^{n(n-1)2}\left(Z_{3i,k|k-1}Z_{3i,k|k-1}^{T}+Z_{4i,k|k-1}Z_{4i,k|k-1}^{T}+Z_{5i,k|k-1}Z_{5i,k|k-1}^{T}+Z_{6i,k|k-1}Z_{6i,k|k-1}^{T}\right)$$

(6) Estimate the cross-covariance matrix:

(48) $$P_{xz,k|k-1}=w_{0}X_{0i,k|k-1}Z_{0i,k|k-1}^{T}+P_{xz1}+P_{xz2}-{\hat{x}}_{k|k-1}{\hat{z}}_{k|k-1}^{T}$$

(49) $$P_{xz1}=w_{1}\sum_{i=1}^{n}\left(X_{1i,k|k-1}Z_{1i,k|k-1}^{T}+X_{2i,k|k-1}Z_{2i,k|k-1}^{T}\right)$$

(50) $$P_{xz2}=w_{2}\sum_{i=1}^{n(n-1)/2}\left(X_{3i,k|k-1}Z_{3i,k|k-1}^{T}+X_{4i,k|k-1}Z_{4i,k|k-1}^{T}+X_{5i,k|k-1}Z_{5i,k|k-1}^{T}+X_{6i,k|k-1}Z_{6i,k|k-1}^{T}\right)$$

(7) Estimate the Kalman gain:

(51) $$W_{k}=P_{xz,k|k-1}P_{zz,k|k-1}^{-1}$$

(8) Estimate the updated state:

(52) $${\hat{x}}_{k|k}={\hat{x}}_{k|k-1}+W_{k}\left(z_{k}-{\hat{z}}_{k|k-1}\right)$$

(9) Estimate the corresponding error covariance:

(53) $$P_{k|k}=P_{k|k-1}-W_{k}P_{zz,k|k-1}W_{k}^{T}$$

## 3. IMM High Degree Cubature Kalman Filter

**Figure 1:**
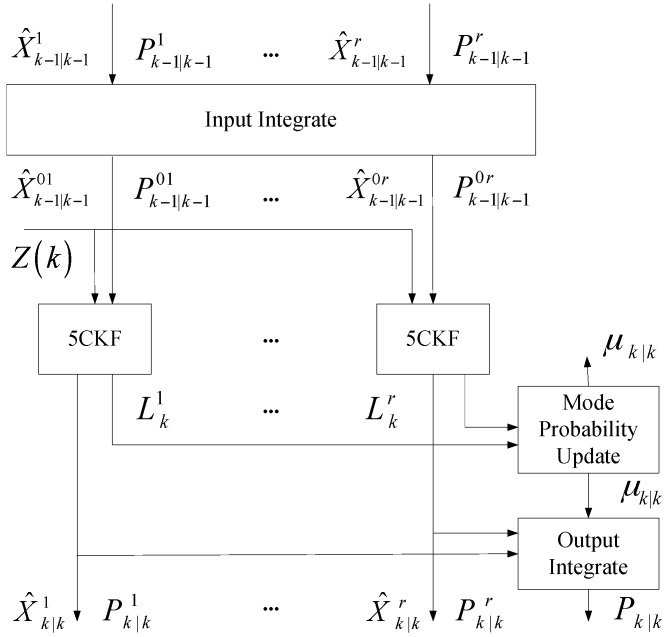
IMM-5CKF structure diagram.

In the paper, the proposed IMM5CKF includes the merits of the 5CKF algorithm and IMM algorithm. The main character of IMM5CKF is that it calculates the state distribution and error covariance matrix by choosing an odd number of special cubature points with equal weights, and the negative weights go to 0 when the dimension of the system goes to ∞. This means that it is more stable than UKF. The IMM-5CKF algorithm includes input integration, five degree cubature Kalman filter, model probability update and output integration. The structure diagram is shown in Figure 1. The filtering processes are shown in the following subsection.

### 3.1. Input Integration

(54) $$u_{k-1|k-1}^{i/j}=\frac{p_{ij}u_{k-1}^{i}}{C_{j}}$$

(55) $${\hat{X}}_{k-1|k-1}^{0j}=\sum_{}^{}{\hat{X}}_{k-1|k-1}^{i}u_{k-1|k-1}^{i/j}$$

(56) $$P_{k-1|k-1}^{0j}=\sum_{i=1}^{r}u_{k-1|k-1}^{i/j}\left\{P_{k-1|k-1}^{i}+\left[{\hat{X}}_{k-1|k-1}^{i}-{\hat{X}}_{k-1|k-1}^{0j}\right]{\left[{\hat{X}}_{k-1|k-1}^{i}-{\hat{X}}_{k-1|k-1}^{0j}\right]}^{T}\right\}$$

where $C_{j}=\sum_{i=1}^{r}p_{ij}u_{k-1}^{i}$, $u_{k-1|k-1}^{i/j}$ is the conditional probability of model i at time k−1, $u_{k-1}^{i}$ is the probability of model i at time k−1, ${\hat{X}}_{k-1|k-1}^{0j}$ is the initial mean value of model j, $P_{k-1|k-1}^{0j}$ is the initial error covariance, ${\hat{X}}_{k-1|k-1}^{i}$ is the estimated value of model i at time k−1, $P_{k-1|k-1}^{i}$ is the relative covariance.

### 3.2. Five Degree Cubature Kalman Filtering

The mixed initial value and measure value (z) are the input of each filter at time k. Then the new state vector ${\hat{X}}_{k|k}^{j}$, error covariance $P_{k|k}^{j}$, predicted measure value $z_{k|k-1}^{j}$ and residual $v_{k}^{j}$ can be got from the 5CKF.

The likelihood value $L_{k}^{j}$ is:

(57) $$L_{k}^{j}=N\left(z;z_{k|k-1}^{j},v_{k}^{j}\right)=\frac{1}{\sqrt{2\pi V_{k}^{j}}}\cdot \exp\left(-\frac{1}{2}{\left[z_{k}-{\hat{z}}_{k|k-1}^{j}\right]}^{T}{\left(V_{k}^{j}\right)}^{-1}\left[z_{k}-{\hat{z}}_{k|k-1}^{j}\right]\right)$$

where $V_{k}^{j}$ is the associated covariance of residual $v_{k}^{j}$.

### 3.3. Model Probability Update

It has been known that if the filter model matches with the actual model, the filter residual is zero and the variance v(k) is Gaussian White Noise. Then the model probability can be updated by Equation (58):

(58) $$u_{k}^{j}=\frac{L_{k}^{j}C_{j}}{\sum_{j=1}^{n_{m}}L_{k}^{j}C_{j}}$$

### 3.4. Output Integration

The probabilities of the model are integrated with the estimated value of each filter based on the given weights. The output of IMM-5CKF can be calculated as:

(59) $${\hat{X}}_{k|k}=\sum_{j=1}^{r}X_{k|k}^{j}u_{k}^{j}$$

(60) $$P_{k|k}=\sum_{j=1}^{r}u_{k}^{j}\left\{P_{k|k}^{j}+\left[{\hat{X}}_{k|k}^{j}-{\hat{X}}_{k|k}\right]{\left[{\hat{X}}_{k|k}^{j}-{\hat{X}}_{k|k}\right]}^{T}\right\}$$

## 4. Results and Discussion

In this section, the IMM-5CKF is compared with IMMCKF, IMMUKF, 5CKF and OMTM-IMM in a benchmark target tracking scenario. The state variable at time k is $X_{k}={\left[x,\dot{x},y,\dot{y}\right]}^{T}$, where x and y are the position variables, $\dot{x}$ and $\dot{y}$ are the velocity variables.

The coordinated turn model is:

(61) $$F2=\left[\begin{array}{cccc} 1 & \frac{\sin\left(\omega T\right)}{\omega } & 0 & \frac{\left(\cos\left(\omega T\right)-1\right)}{\omega }\\ 0 & \cos\left(\omega T\right) & 0 & -\sin\left(\omega T\right)\\ 0 & \frac{\left(1-\cos\left(\omega T\right)\right)}{\omega } & 1 & \frac{\sin\left(\omega T\right)}{\omega }\\ 0 & \sin\left(\omega T\right) & 0 & \cos\left(\omega T\right) \end{array}\right]$$

where ω is the turn rate and T is the sampling interval. The right turn rate is defined as −3°, and the left turn rate is 3°.

The measurement equation of the system is:

(62) $$Z=\left[\begin{array}{cccc} 1 & 0 & 0 & 0\\ 0 & 0 & 1 & 0 \end{array}\right]+R$$

where R is the measurement noise of the system.

The initial state $X_{0}={\left[1000\;m,200\text{ m/s},1000\;m,200\text{ m/s}\right]}^{T}$, initial associate covariance is $P_{0}=diag\left(\left[1000,10,1000,10\right]\right)$, process noise Q~N(0,q), q=[10,0;0,10], process noise weight matrix is $G=\left[\frac{T^{2}}{2},0;T,0;0,\frac{T^{2}}{2};0,T\right]$. The measure noise R~N(0,r), with r=diag([20,0.1]).

The simulation time simTime=100s, the step time T=1 s. The target turns right during 20 s~40 s, turns left during 60 s~80 s, and maintains uniform motion during the other time. The model transition probability is:

(63) $$p_{ij}=\left[\begin{array}{ccc} 0.9 & 0.05 & 0.05\\ 0.1 & 0.8 & 0.1\\ 0.05 & 0.15 & 0.8 \end{array}\right]$$

The root-mean square error (RMSE) of position and velocity are used to contrast the performance of the filtering algorithms. The RMSE defined in state vector X at k is:

(64) $$RMSE=\sqrt{\frac{\sum_{n=1}^{n}\frac{1}{k}{\sum_{k=1}^{k}\left(X_{k,n}-{\hat{X}}_{k,n}\right)}^{2}}{n}}$$

**Figure 2:**
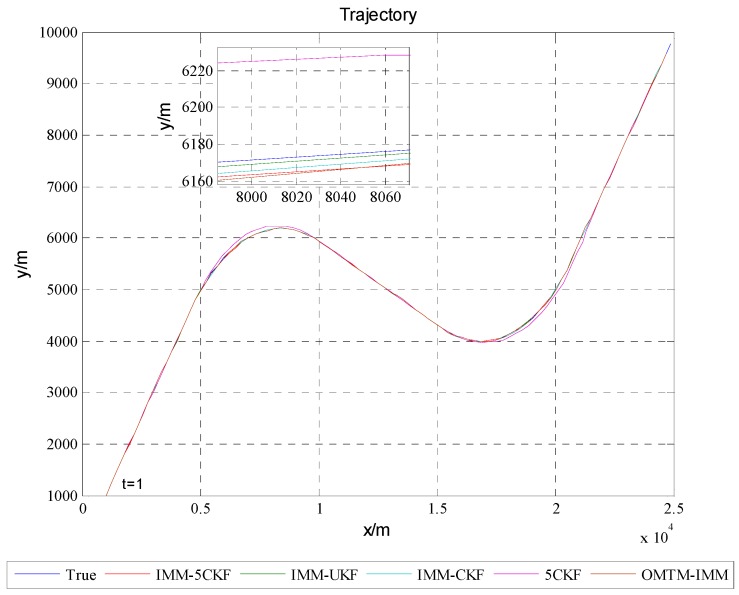
Target Trajectory.

**Figure 3:**
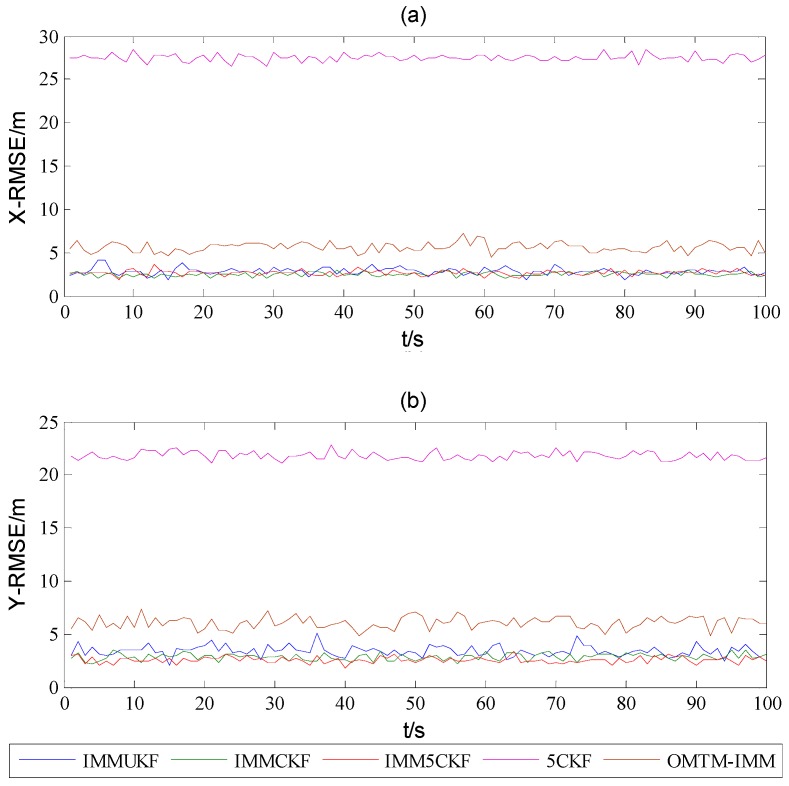
RMSEs of (**a**) X-position and (**b**) Y-position.

**Figure 4:**
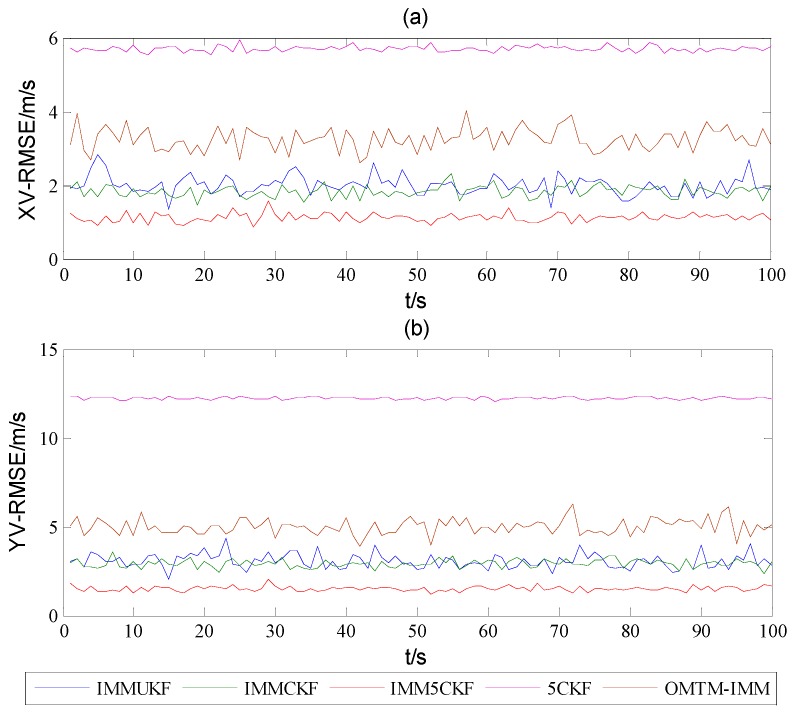
RMSEs of (**a**) X-velocity and (**b**) Y-velocity.

Figure 2 shows the target trajectory after 100 Monte Carlo simulations, from which it can be found that all the algorithms could track the trajectory of the target. Figure 3 and Figure 4 show that the estimated RMSEs in position and velocity of IMM5CKF, IMMCKF, IMMUKF, 5CKF and OMTM-IMM respectively. From Figure 3 and Figure 4, it can be found that all the algorithms exhibit stable characteristics, and there are no error divergence during the simulation time. In addition, the results show that the RMSEs of IMM5CKF are less than those of the other algorithms and the performance is more stable. In Figure 3 and Figure 4, the RMSEs of 5CKF is the largest, which means a single model algorithm cannot adapt to changeable target tracking problems. In [21], authors had proved that OMTM-IMM performs better than traditional IMM algorithm. In Figure 3 and Figure 4, it can be seen that the accuracy of OMTM-IMM is better than 5CKF, but worse than that of the other algorithms which are based on improved nonlinear filters.

The RMSEs of the IMM5CKF, IMMCKF, IMMUKF, 5CKF and OMTM-IMM are shown in Table 1. The data shows that the tracking accuracy of IMM-5CKF is better than that of the other algorithms with increasing computational load.

**Figure 5:**
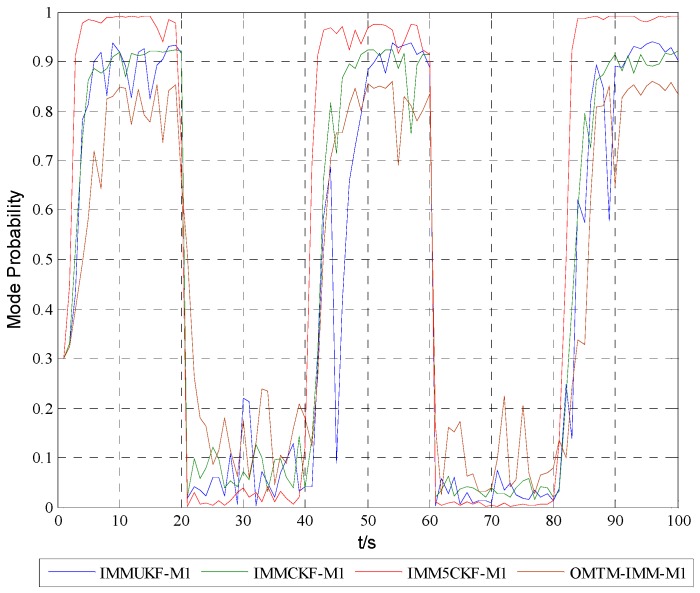
Model probabilities of model 1.

**Figure 6:**
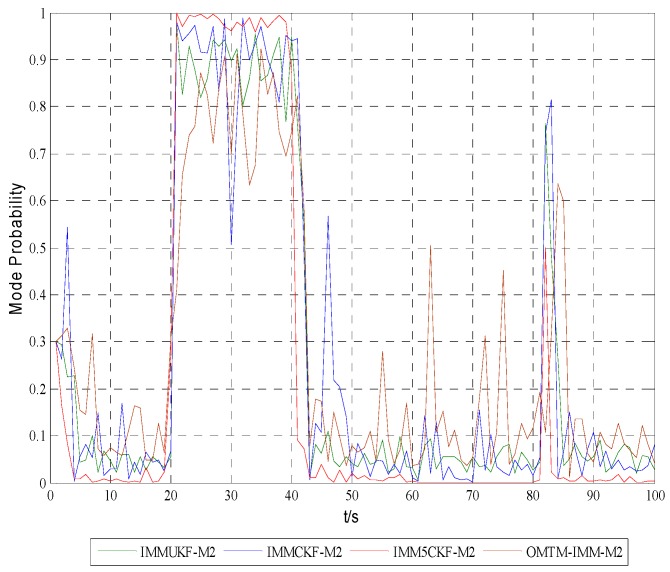
Model probabilities of model 2.

**Figure 7:**
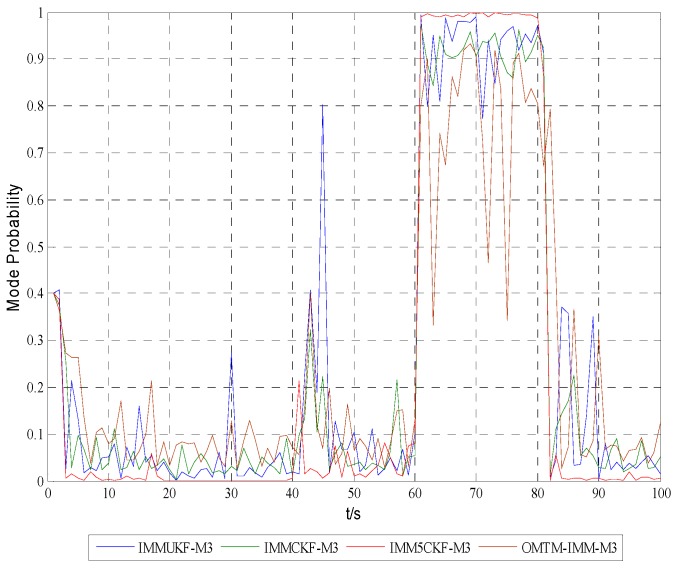
Model probabilities of model 3.

Figure 5, Figure 6 and Figure 7 show the model probabilities of IMM5CKF, IMMCKF, IMMUKF and OMTM-IMM.

Figure 5, Figure 6 and Figure 7 demonstrate that IMM5CKF, IMMCKF, IMMUKF and OMTM-IMM can effectively track the model characteristics of a maneuvering target. It is also found that the IMM-5CKF can capture the kinematics of maneuvering in time once the motion state changes at time t=20 s, t=40 s, t=60 s and t=80 s. The simulation results show that IMM5CKF has an obvious advantage over the other algorithms in target tracking problems.

## 5. Conclusions

In this paper, IMM5CKF is proposed to enhance the tracking accuracy, model estimation accuracy and response sensitivity of nonlinear maneuvering target tracking problems. The algorithm introduces a five degree cubature Kalman filter into interacting multiple models which simultaneously disposes of all the models through a Markov Chain. A classical target tracking problem is utilized to demonstrate that the IMM5CKF can indeed improve the quick response sensitivity of target tracking algorithm, and it exhibits more accurate than IMMCKF, IMMUKF, CKF and OMTM-IMM. In our future research, the study may focus on multisensor navigation and positioning systems. The proposed algorithm should be suitable for the complex real environments according to the analysis.

## Figures and Tables

**Table 1:** The RMSEs of the different target tracking algorithms.

RMSE	IMM5CKF	IMMCKF	IMMUKF	5CKF	OMTM-IMM
RMSE_X (m)	2.6675	2.4847	2.5392	27.4975	5.6211
RMSE_X_V (m/s)	1.1245	1.8306	1.8930	5.7001	3.2510
RMSE_Y (m)	2.5255	2.8534	3.0362	21.7947	6.0674
RMSE_Y_V (m/s)	1.4972	2.9201	2.8488	12.2331	4.9938
Time (s)	14.9726	7.2549	7.3785	5.3101	6.0314

## References

[1] Read J., Achutegui K., Miguez J. (2014). A distributed particle filter for nonlinear tracking in wireless sensor networks. Signal Process..

[2] Li W., Jia Y., Du J., Zhang J. (2014). PHD filter for multi-target tracking with glint noise. Signal Process..

[3] Meyer F., Hlinka O., Hlawatsch F. (2014). Sigma point belief propagation. IEEE Signal Process. Lett..

[4] Zhang T., Wu R. (2015). Affinity propagation clustering of measurements for multiple extended target tracking. Sensors.

[5] Morelande M.R., Garcia-Fernandez A.F. (2013). Analysis of Kalman filter approximations for nonlinear measurements. IEEE Trans. Signal Process..

[6] Bugallo M.F., Xu S., Djurić P.M. (2007). Performance comparison of EKF and particle filtering methods for maneuvering targets. Digit. Signal Process..

[7] Jwo D.J., Wang S.H. (2007). Adaptive fuzzy strong tracking extended Kalman filtering for GPS navigation. IEEE Sens. J..

[8] Frogerais P., Bellanger J.J., Senhadji L. (2012). Various ways to compute the continuous–discrete extended Kalman filter. IEEE Trans. Autom. Control.

[9] Julier S., Uhlmann J., Durrant-Whyte H.F. (2000). A new method for the nonlinear transformation of means and covariances in filters and estimators. IEEE Trans. Autom. Control.

[10] Arasaratnam I., Haykin S. (2009). Cubature Kalman filters. IEEE Trans. Autom. Control.

[11] Arasaratnam I., Haykin S., Hurd T.R. (2010). Cubature Kalman filtering for continuous–discrete systems: Theory and simulations. IEEE Trans. Signal Process..

[12] Sun F., Tang L.J. (2013). Estimation precision comparison of Cubature Kalman filter and Unscented Kalman filter. Control Decis..

[13] Blom H.A., Bar-Shalom Y. (1998). The interacting multiple model algorithm for systems with markovian switching coefficients. IEEE Trans. Autom. Control.

[14] Cui N., Hong L., Layne J.R. (2005). A comparison of nonlinear filtering approaches with an application to ground target tracking. Signal Process..

[15] Gao L., Xing J., Ma Z., Sha J., Meng X. (2012). Improved IMM algorithm for nonlinear maneuvering target tracking. Procedia Eng..

[16] Zhang Y., Guo C., Hu H., Liu S., Chu J. (2014). An Algorithm of the Adaptive Grid and Fuzzy Interacting Multiple Model. J. Mar. Sci. Appl..

[17] Li W., Jia Y. (2012). Location of mobile station with maneuvers using an IMM-based cubature Kalman filter. IEEE Trans. Ind. Electron..

[18] Wan M., Li P., Li T. Tracking maneuvering target with angle-only measurements using IMM algorithm based on CKF. Proceedings of the 2010 International Conference on Communications and Mobile Computing.

[19] Lee S.J., Motai Y., Choi H. (2013). Tracking human motion with multichannel interacting multiple model. IEEE Trans. Ind. Inform..

[20] Himberg H., Motai Y., Bradley A.P. (2013). A multiple model approach to tracking head orientation with delta quaternions. IEEE Trans. Cybern..

[21] Barrios C., Motai Y., Huston D. (2015). Intelligent forecasting using dead reckoning with dynamic errors. IEEE Trans. Ind. Inform..

[22] Zhou W., Cai J., Sun L., Shen C. (2014). An improved interacting multiple model algorithm used in aircraft tracking. Math. Probl. Eng..

[23] Jia B., Xin M., Cheng Y. (2013). High-degree cubature Kalman filter. Automatica.

